# Design of Novel Haptens and Development of Monoclonal Antibody-Based Immunoassays for the Simultaneous Detection of Tylosin and Tilmicosin in Milk and Water Samples

**DOI:** 10.3390/biom9120770

**Published:** 2019-11-23

**Authors:** Jian-Xin Huang, Chan-Yuan Yao, Jin-Yi Yang, Zhen-Feng Li, Fan He, Yuan-Xin Tian, Hong Wang, Zhen-Lin Xu, Yu-Dong Shen

**Affiliations:** 1 College of Food Science, Guangdong Provincial Key Laboratory of Food Quality and Safety, South China Agricultural University, Guangzhou 510642, China; bi@stu.scau.edu.cn (J.-X.H.); ciel32o@163.com (C.-Y.Y.); yjy361@163.com (J.-Y.Y.); snowywhite@163.com (F.H.); gzwhongd@163.com (H.W.); jallent@163.com (Z.-L.X.); 2 Department of Entomology and Nematology and UCD Comprehensive Cancer Center, University of California Davis, Davis, CA 95616, USA; fzhli@ucdavis.edu; 3 Guangdong Provincial Key Laboratory of New Drug Screening, School of Pharmaceutical Sciences, Southern Medical University, Guangzhou 510515, China

**Keywords:** hapten, monoclonal antibody, immunoassay, tylosin, tilmicosin

## Abstract

In this work, a monoclonal antibody-based indirect competitive enzyme-linked immunosorbent assay (icELISA) was established to detect tylosin and tilmicosin in milk and water samples. A sensitive and specific monoclonal antibody was prepared by rational designed hapten, which was achieved by directly oxidizing the aldehyde group on the side chain of tylosin to the carboxyl group. Under the optimized conditions, the linear range of icELISA for tylosin and tilmicosin were 1.3 to 17.7 ng/mL and 2.0 to 47.4 ng/mL, with half-maximal inhibition concentration (IC_50_) values of 4.7 and 9.6 ng/mL, respectively. The cross-reactivity with other analogues of icELISA was less than 0.1%. The average recoveries of icELISA for tylosin and tilmicosin ranged from 76.4% to 109.5% in milk and water samples. Besides, the detection results of icELISA showed good correlations with HPLC-MS/MS. The proposed icELISA was satisfied for rapid and specific screening of tylosin and tilmicosin residues in milk and water samples.

## 1. Introduction

**Figure 1:**
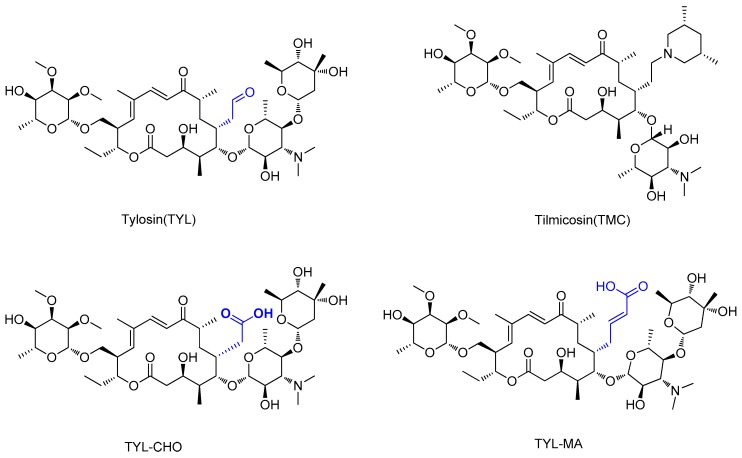
The structures of tylosin (TYL), tilmicosin (TMC), and haptens (TYL-CHO and TYL-MA). The spacer arm of two haptens labeled in blue.

Tylosin is a macrolide antibiotic obtained from the culture fluid of *Streptomyces fradiae* and has a wide antibacterial spectrum against gram-positive organisms, selected gram-negative organisms, as well as *mycoplasma*, *Rickettsia*, and *Chlamydia* [1,2]. Tilmicosin is the semisynthetic derivative of tylosin (Figure 1), which has a similar antibacterial spectrum to tylosin [3]. Furthermore, tilmicosin has a better antibiosis effect than tylosin against gram-negative organisms, *mycoplasma*, *pasteurella*, and can be used to control mycoplasma avian disease and mastitis of lactating cows [4,5,6]. Due to effective antibiotic properties, tylosin and tilmicosin are widely applied to treat bacterial infection in various animals, including beef cattle swine and poultry [4,7]. Besides, tylosin and tilmicosin can also promote animal growth and increase feed utilization as a feeding additive [8,9]. Tylosin and tilmicosin have a low metabolic rate after administration, and their residues are widely distributed in the body fluids and tissues of livestock [7,10]. In addition, it was reported that tylosin and tilmicosin residues could also be tested in environmental samples, such as water [11,12]. Therefore, excessive use of these antibiotics could result their residues existing in animal tissues or the environment, and intake with prolonged low doses of antibiotics can bring potential risk to human health, such as human allergy and resistance [13,14,15]. Thus, maximum residue limits (MRLs) for tylosin and tilmicosin have been established as 0.05 to 1.5 mg/kg in food samples in China [16], and banned as feed additives in the European Union [17].

Currently, methods for simultaneous detection of tylosin and tilmicosin mainly include high-performance liquid chromatography (HPLC), high performance liquid chromatography coupled with tandem mass spectrometry (HPLC-MS/MS), and other large-scale chromatographic methods [18,19,20]. These large-scale equipment methods are highly sensitive and accurate, but are expensive, time-consuming, have low sample throughput, and are inconvenient for detection in the field. As an alternative, due to its advantages of being fast, easy to use, low cost, and portable, the most practical immunoassay, primarily enzyme-linked immunosorbent assay (ELISA), has been widely developed and used to rapidly monitor various pesticides and veterinary drugs in food and environment samples with high-throughput sample, and plays a vital role in field detection [21,22,23,24]. It is well known that antibodies are the core reagents for the development of immunoassay methods. To date, the antibodies used in the simultaneous detection of tylosin and tilmicosin are mainly obtained by immunization with Shiff base arm hapten derived from carbonyl amino condensation and some of these reported immunoassays are also sensitive and accurate [2,25,26,27,28]. Thus, our first attempt was to use the reported Shiff base hapten strategy, but this failed to obtain an antibody against tylosin and tilmicosin because of the poor and unstable immune response. We speculated that the lone spacer arm attached to the tylosin molecules could change the original conformation of tylosin, and the carbon–nitrogen double bond arm in the reported Shiff base hapten could be instable for long-term maintenance of the immune response in animals [29]. So, as an alternative attempt, novel hapten strategies for mimicking tylosin to the maximum extent were proposed for steadily inducing an antibody against tylosin and cross-reacted with tilmicosin. Finally, the icELISA method was developed for the simultaneous detection of tylosin and tilmicosin in milk and water samples in this study.

## 2. Materials and Methods

### 2.1. Materials and Instruments

Tylosin, tilmicosin, bovine serum albumin (BSA), spiramycin, roxithromycin, avermectin, N-Hydroxysuccinimide (NHS), dicyclohexylcarbodiimide (DCC), ovalbumin (OVA), HAT Media Supplement (50×), complete and incomplete Freund’s adjuvant, goat anti-mouse Immunoglobulin G (IgG) horseradish peroxidase conjugate (HRP-IgG), and Tween-20 were purchased from Sigma-Aldrich (St. Louis, MO, USA). Acetylspramycin, furadantin, enrofloxacin, pyridine, and triethylamine were purchased from Shanghai Civic Chemical Technology Co., Ltd. (Shanghai, China). Nitrofural, erythromycin, azithromycin, pefloxacin, and propanedioic acid were purchased from Shanghai Macklin Biochemical Co., Ltd. Fetal calf serum, Pierce Rapid Isotyping Kits-Mouse, poly-ethylene glycol (PEG-1450), and RPMI Medium 1640 basic (1×) were purchased from Thermo Fisher Scientific Co., Ltd. (Waltham, MA, USA). Protein-G Resin was purchased from TransGen Biotech. Inc. (Beijing, China). Other chemicals were purchased from Guangzhou chemical reagent Co., Ltd. Female BALB/c mice were purchased from Guangdong Medical Laboratory Animal Center (Guangzhou, China). Phosphate solution (PBS, 0.01 M at pH 7.4): 8.5 g/L NaCl, 2.9 g/L Na_2_HPO_4_·12H_2_O, 0.2 g/L KH_2_PO_4_, 0.2 g/L KCl). Dilution solution (0.01 M PBST at pH 7.4): 0.1% Tween-20 in 0.01 M PBS. Coating buffer: 0.1 M carbonate buffer at pH 9.6. Blocking buffer: 0.05% Tween-20 and 5% skim milk powder in 0.01 M PBS. Washing solution: 8.5 g/L NaCl, 2.9 g/L Na_2_HPO_4_·12H_2_O, and 0.01% Tween-20. Stop solution: 20% sulfuric acid. Nano Drop 2000C ultra-violet spectrophotometer (Thermo Scientific, Waltham, MA, USA), DEM-3 automatic plate washer (Top Analytical Instruments Co., Ltd., Beijing, China), AB SCIEX 5500 triple quadrupole mass spectrometer (AB SCIEX, Redwood City, CA, USA), and Hypersil GOLD-C18 (2.1 mm × 100 mm, 1.9 μM, Thermo Scientific, Waltham, MA, USA) were used in this study.

### 2.2. Synthesis of Haptens and Antigens

Synthesis of TYL-CHO. The hapten TYL-CHO was obtained by using our own previously reported methods [30]. Tylosin (1 g, 1.09 mmol) was dissolved in 80 mL of 1-Butanol: H_2_O (5:3, *v*/*v*) at 0 °C. NaH_2_PO_4_ (0.62 g, 5.17 mmol), 1 mL of dimethyl sulfoxide, and NaClO_2_ (0.2 mg, 2.21 mmol) were added in cold solution sequentially and refluxed for 30 min. The mixture was extracted by ethyl acetate/brine, and then the organic layer was dried over anhydrous Na_2_SO_4_. After removal of solvent under reduced pressure, the crude mixture was purified by column chromatography (MeOH:Trichloromethane = 1:10, *v*/*v*), and hapten TYL-CHO was obtained (Figure S1A) and confirmed by ESI-MS (*m*/*z*, positive, 932.5494 [M + H]^+^).

Synthesis of TYL-MA. Tylosin (1 g, 1.09 mmol) and propanedioic acid (0.1 g, 0.96 mmol) were dissolved in 10 mL of pyridine. In total, 1 mL of triethylamine was added in solution and refluxed under nitrogen for 2 h. The mixture was added in 2 M hydrochloric acid aqueous solution and cooled until room temperature. After filtration, the deposit was washed by water and recrystallized by ethanol aqueous solution. The crude mixture was purified by column chromatography (MeOH:Trichloromethane = 1:10, *v*/*v*), and TYL-MA was obtained (Figure S1B) and confirmed by ESI-MS (*m*/*z*, positive, 958.5473 [M + H]^+^).

The two haptens were conjugated with BSA for use as immunizing conjugates and OVA as coating conjugates according to the modified procedure in [31]. Hapten (0.1 mmol), DCC (0.2 mmol), and NHS (0.2 mmol) were dissolved in 0.5 mL of DMF and stirred at 4 °C overnight. In total, 15 mg of BSA or OVA were dissolved in 5 mL of 0.1 M PBS (pH = 7.4). Next, 250 μL of the hapten reaction mixture were added to the carrier protein solution slowly and stirred at 4 °C for 12 h. After being dialyzed in PBS at 4 °C for 3 days, the mixture was stored at −20 °C until use. The structure of the conjugate was confirmed by UV-vis spectral.

### 2.3. Immunization and Monoclonal Antibodies

Each female BALB/c mouse (7–8 weeks old) was immunized with immunogen that was prepared by emulsification of immunogen (1 mg/mL, 100 μL) and Freund’s adjuvant (100 μL). The immunizing conjugates were injected subcutaneously on the back and abdomen of each mouse. Complete adjuvant was used for the first injection. After three weeks, the incomplete adjuvant was used for the subsequent injections in every two weeks. One week after the fourth injection, mice sera were collected from their tail tip and evaluated by icELISA and the mice with the best response was chose for the generation of hybridomas by cell fusion.

Myeloma cells Sp2/0 were fused with the spleen cells of immunized mice at a ratio of 5:1 according to the modified procedure of [32]. Hybridomas from wells with a positive response by icELISA were selected. After cell culture, the hybridomas were injected into mice intraperitoneally and the ascites were collected after 8 to 10 days. The ascites was purified by column chromatography of Protein-G Resin. The purified monoclonal antibodies (mAbs) were detected as the concentration of the antiserum by NanoDrop 2000c and stored at −20 °C until use.

### 2.4. icELISA Protocol

An icELISA was used to evaluate the binding ability and specificity of sera or monoclonal antibody (mAb). Serial concentrations of the coating antigens in coating buffer were added in the 96-well polystyrene ELISA plates with 100 μL per wells and incubated at 37 °C overnight. Then, the wells were washed two times with washing buffer and the uncoated sites were blocked for 3 h at 37 °C. Finally, the plates were dried at 37 °C for 1 h and stored at 4 °C until use. Briefly, 50 μL of varying concentration sera or mAb in 0.01 M PBST and 50 μL of varying concentration of tylosin standard were incubated in plates at 37 °C for 40 min. Then, the plates were washed five times and 100 μL of HRP-anti mouse IgG were added in PBST (1:5000) at 37 °C for 30 min. After being washed five times, 100 μL of substrate solution were added in the plates and incubated at 37 °C for 10 min. Finally, by the addition of stop solution, with 50 μL added to each well, the optical density was measured at 450 nm by Multiskan MK3. The percentage inhibition of sera or mAb binding ability was expressed as follows: Titer means the dilution of antiserum with an absorbance at 450 nm of about 1.0 to 1.5, with a high inhibition (inhibition = [(B − B_0_)/B] × 100%, B means the absorbance without a competitor and B_0_ means the absorbance with a competitor).

### 2.5. Optimization of icELISA Condition

To improve the sensitivity of icELISA, several parameters were optimized, including the concentration of antibody, coating antigens and HRP-IgG, working buffer (ionic strength, pH value and the concentration of Tween-20), and reaction time. According to the dose–response curves, the maximum absorbance value (A_max_) and half maximal inhibitory concentration (IC_50_) were calculated. The optical conditions were confirmed by A_max_, A_max_/IC_50_, and IC_50_. A condition with a higher value of A_max_/IC_50_ and lower value of IC_50_ were selected to use in the assay. After the optimization of condition, serial dilutions of tylosin or tilmicosin were added in the working buffer as the competitor to construct the standard curve by sigmoidal curve fitting in origin pro 9.0 software (Originlab Corp., Northampton, MA, USA).

### 2.6. Preparation and Elimination of Matrix Effect in Samples for icELISA

All the tylosin- and tilmicosin-free samples were confirmed by HPLC-MS/MS and pretreated using the method as follows. For icELISA: 1 mL of milk was dissolved in 20 μL of H_2_SO_4_ (2 mol/L, to remove the fat and protein) and stirred. Then, the mixture was centrifuged at 4000 rpm for 5 min to obtain the supernatant. After being filtered by 0.22 μm of membrane, the supernatant was diluted 2-, 5-, and 10-fold with PBST. Water sample: 1 mL of tap water, drinking water, and environmental water (come from a pond in Guangzhou) were filtered by 0.22 μm of membrane. The tap water, drinking water, and environmental water were diluted 0-, 1-, 2-, 3-, and 5-fold with PBST. Serial dilutions of tylosin or tilmicosin were added in the serial dilution of sample buffers as the competitor to test using icELISA, and compared with the standard curves or visual result constructed by PBST under the optimization condition. The best dilution to eliminate the matrix effect was confirmed by comparing the calibration curves of the diluted sample solution and PBST.

### 2.7. Comparison of icELISA with HPLC-MS/MS

Tylosin or tilmicosin in different concentrations were spiked in the milk and water samples. According to the modified procedure of the icELISA as above, all the spiked samples were subjected by icELISA to test the recovery, and validated by HPLC-MS/MS by referring to the China National Standard [33] with a column of Hypersil GOLD-C18 (2.1 mm × 100 mm, 1.9 μm). The flow rate of the HPLC-MS/MS method was 0.5 mL/min, with a 30 °C column temperature and 10-μL sample size. The mobile phases were 0.1% of formic acid (A) and methanol (B). The gradient elution: 0–25 min, 30–95% of B; 2.5–3 min, 95% of B; 3–3.1 min, 95%–30% of B; and 3.1–5 min, 30% of B.

Linear regression between the method of icELISA and HPLC-MS/MS was used to check the consistency of these methods. To testify the verification of this icELISA method, the actual samples were selected randomly and analyzed by icELISA and HPLC-MS/MS. In total, 40 blind samples were purchased or provided from local supermarkets, Guangdong institute for food inspection, ponds, laboratory, and household tap in Guangzhou.

### 2.8. Molecular Simulation

Molecule modelling was performed by Schrodinger software package. The chemical structures of haptens, TYL, and TMC were constructed by 2D sketcher. Their geometries were optimized by OPLS3 force field implemented in Schrodinger, Maestro 11.1. Then, they were aligned by the quick align method. The electrostatic potential of selected difference atoms were colored in the surface. The color ramp was red_white_blue.

## 3. Results and Discussion

### 3.1. Design and Screening of the Haptens

**Figure 2:**
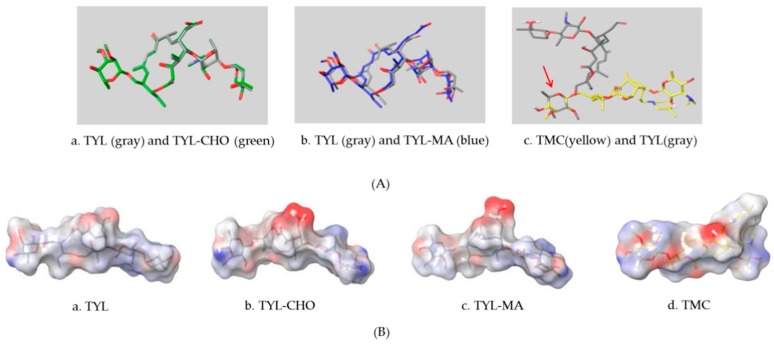
The alignment and electrostatic potential isosurfaces of haptens and target. (**A**) The structural alignment view, the red arrow point to the overlapping group between TYL and TMC; (**B**) The electrostatic potential isosurfaces, the blue areas indicate positive potential, the white areas indicate neutral potential, and the red areas indicate negative potential.

High-quality antibodies are key for immunoassays, ultimately depending on rational hapten design [34]. Generally, a rational-designed hapten should be highly overlapped with the target molecules in a three-dimensional structure and bring stable immunogenicity to induce an immune response. Besides, monoclonal antibody has a more widespread application than polyclonal antibody for its high specificity and good repeatability according to our ongoing and in-depth research on the development of various immunoassay methods [35,36,37]. Based on our previous studies on polyclonal antibody against tylosin [30], we think that the new trend of antibody production of TYL and TMC should be characterized in terms of sensitivity and specificity, so we attempted to use different hapten strategies to immunize BALB/c mice for the preparation and screening of a high-quality monoclonal antibody. It was reported that more similar the characteristics of a hapten derivative are to the target, such as size, shape (geometry) and electronic properties, the more likely specific antibodies will be produced [38,39]. Moreover, it is also suggested that a short semi-rigid unsaturated double bond structure is generally good for the production of desired antibodies [40,41,42]. Based on these ideas, haptens with short spacer arms or unsaturated double bond arms were designed and the similarity between drugs and haptens was analyzed by molecular simulation. As shown in Figure 1, the spacer of hapten TYL-CHO contained a one-carbon-length carboxyl group, while hapten TYL-MA had a semi-rigid unsaturated spacer that was two carbon atoms longer. The results showed that hapten TYL-CHO gave the most effective specific immune response under the conditions of homology coating and the hapten TYL-MA gave no immune response against tylosin and tilmicosin (Table 1). The difference of the haptens, TYL, and TMC are shown in Figure 2. It is obvious that the hapten TYL-CHO (green) was highly similar to TYL (gray), while the hapten TYL-MA (blue) was quite different from TYL (Figure 2A-a,A-b). The long-chain carboxyl group with a carbon–carbon double bond affected the conformation of the macrocycle, which led to poor alignment with TYL. Furthermore, their electrostatic potential also displayed a remarkable difference (Figure 2B). The hapten TYL-MA is more negative than TYL (red area). Besides, the negative area of hapten TYL-MA was far from the macrocycle, probably responsible for the poor specific immune responses against TYL. Combining the immune results with structure analysis, we speculated that TYL-CHO maintained the spatial structure and electronic properties of tylosin so that the molecular structure of tylosin during immunization was exposed and stimulated the production of stable and specific antibodies efficiently. In addition, the hapten TYL-CHO also showed a high inhibition for TMC (Table 1). The spatial structure of TYL was different on TMC, but they had similar electronic properties (Figure 2B-d), with the same overlapped side-chain structure (Figure 2A-c, the red arrow point to the overlapping group). The overlapped side-chain structure was far away from the derivatized group of hapten, so the side-chain structure was considered as one of the key binding sites for the antibody and caused the high cross-reactivity (49.0%, Table 2) between TYL and TMC. In summary, it was an efficient strategy for target-derived hapten to be designed rationally through structure simulation, and further confirmed that the well-mimicked hapten TYL-CHO had stable immunogenicity to induce an antibody against tylosin in mice (Table 1).

### 3.2. Preparation and Characterization of Monoclonal Antibodies

According to the immune results (Table 1), the TYL-CHO immunized mice with the highest titer and drug inhibition were selected for cell fusion. The mice spleen cells were fused with SP2/0 myeloma cells by the addition of ploy-ethylene glycol (PEG-1450) at a ratio of 5:1, then feeder cells were added in the cell mixture, and cultured in HAT medium for selection cultivation in a CO_2_ incubator. As fused hybridomas were increased, complete medium was used for cultivation, and the culture supernatant from each well were tested by icELISA. The hybridoma cells with strong positive response were sub-cloned three to four times. At last, four isolated hybridoma cells with a strong positive response against tylosin were selected and obtained as ascites from mice. The monoclonal antibodies were purified from ascites and determined by icELISA (Table 3). Of the four mAbs, L02 showed the best sensitivity to tylosin and cross-reacted with tilmicosin so that mAb L02 was selected for the rest of this study. Furthermore, the isotype of mAb L02 belonged to the IgG2b subtype that was tested by a commercial rapid isotyping kit (Figure S5).

### 3.3. The Standard Curve for icELISA

**Figure 3:**
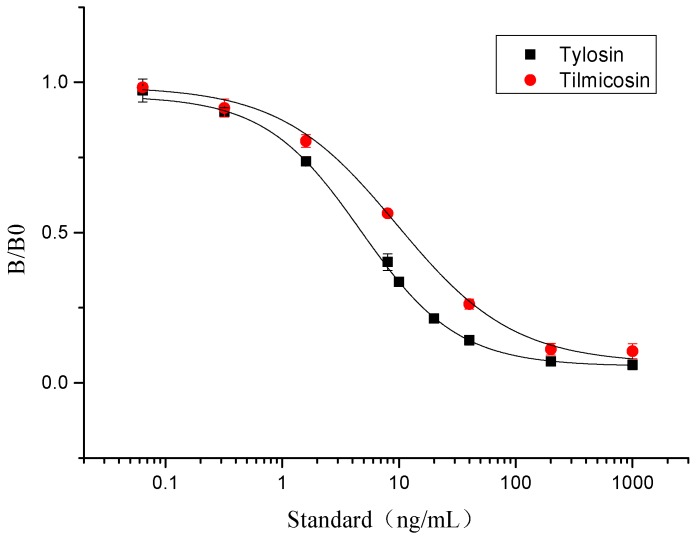
The icELISA standard curves of tylosin and tilmicosin.

Due to the immunoassay being an equilibrium binding reaction, it required a suitable dose of antibody, antigen, secondary antibody, and an appropriate buffer system [43]. The pH of the buffer could directly influence the sensitivity of the assay, and surfactant, such as Tween-20, could increase the hydrophilicity of the standards [43,44]. In this study, the concentration of antibody and antigen were determined by chessboard titration (Table S1). The optimum icELISA conditions were determined as follows (Figure S4): The antibody (L02) was diluted 1:16000 (0.0625 μg/mL) in PBST (0.01 mol/mL, 0.1% of Tween-20, pH 7.4) with 0.11 μg/mL of coating antigen. The HRP-IgG was diluted 1:5000 in 0.01 M PBST and its reaction time with antibody was 30 min. The standard curves of tylosin and tilmicosin were constructed under these conditions (Figure 3). The IC_50_ value of icELISA for tylosin and tilmicosin were 4.7 and 9.6 ng/mL, respectively. The linear range (IC_20_–IC_80_) was 1.3 to 17.7 ng/mL for tylosin and the one for tilmicosin was 2.0 to 47.4 ng/mL. In short, the novel designed hapten TYL-CHO could induce a high sensitivity antibody and be applied for icELISA after optimization.

### 3.4. Specificity of icELISA

The cross-reactivity (CR) of monoclonal antibody L02 with nine macrolides antibiotics or related compounds is summarized in Table 2. The results demonstrated the antibody had cross-reactivities of 49.0% (icELISA) with tilmicosin, and extremely low cross-reactivities (<0.01%) with other macrolides antibiotics and functional compounds, which confirmed that the monoclonal antibody (L02) could detect tylosin and tilmicosin simultaneously with high specificity, confirming the effectiveness of the rationally designed hapten.

### 3.5. Elimination of Matrix Effect in Samples of icELISA

**Figure 4:**
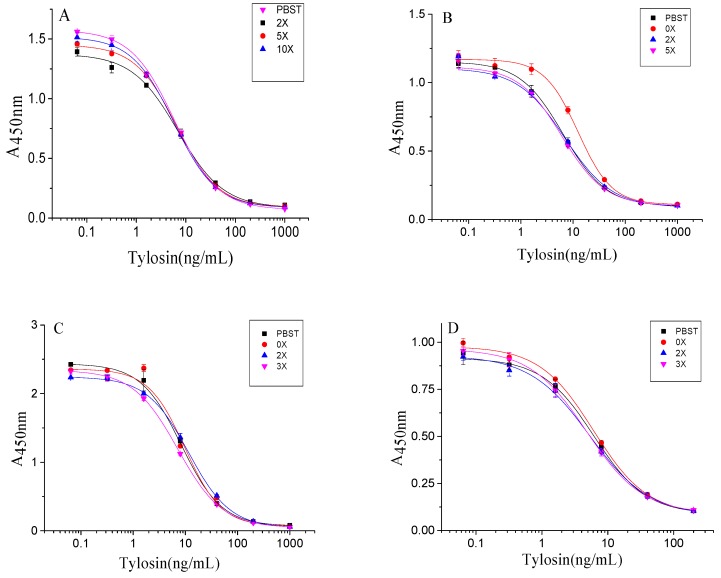
Matrix effect of the samples. ((**A**) milk; (**B**) drinking water; (**C**) tap water; (**D**) environmental water).

The matrix of samples influenced the results of icELISA. The milk contains complex matrix compounds, such as proteins and fats, and the water samples contain invisible impurities and lack ions like sodium and potassium in the buffer system [45]. To eliminate the matrix effect in icELISA (Figure 4), the milk was diluted 1:10 in 0.01 M PBST. The tap water and drinking water were diluted 1:2 in 0.01 M PBST and environmental water was diluted 1:2 in 0.01 M PBST. As a result, this icELISA method could accomplish detection after a simple sample pretreatment.

### 3.6. Test in Spiked Samples and Real Positive Sample

To evaluate the reliability of these results, all tylosin- and tilmicosin-free samples were confirmed by HPLC-MS/MS. Serial concentrations of tylosin and tilmicosin were spiked in the samples and detected the recoveries and coefficient of variance (CV) by icELISA and HPLC-MS/MS, respectively. As illustrated in Table 4, the recoveries of tylosin and tilmicosin were 76.4% to 109.5% for icELISA with CVs less than 15%. Comparison of the results of icELISA and HPLC-MS/MS methods using linear regression analysis (Figure S7) showed there were good correlations between the two methods (R^2^ = 0.93), revealing the good accuracy of icELISA. In addition, there were 11 positive samples in 40 real blind samples, which were confirmed by HPLC-MS/MS, and the analysis of real positive samples by icELISA also had good consistence with the analysis of HPLC-MS/MS (Table 5). In general, this icELISA could be applied for the detection of tylosin and tilmicosin simultaneously in real milk and water samples with high accuracy.

## 4. Conclusions

Based on a new rational hapten design strategy instead of traditional shiff base approach for tylosin and tilmicosin, a sensitive and effective antibody with stable immunogenicity was obtained in this study. After optimization, the icELISA for tylosin and tilmicosin was developed with high sensitivity and specificity. These developed assays were appraised by the cross-reactivity and recovery and validated by the HPLC-MS/MS results. In summary, this icELISA method was satisfactory for screening a large number of milk and water samples rapidly and could be applied for the detection of tylosin and tilmicosin simultaneously to meet different testing requirements.

## Figures and Tables

**Table 1:** Titer and specificity of antiserum.

Immunogen	TYL-CHO-BSA			TYL-MA-BSA		
Coating Antigens	Titer ^a^ (10^3^)	Inhibition ^b^ of TYL (%)	Inhibition of TMC (%)	Titer (10^3^)	Inhibition of TYL (%)	Inhibition of TMC (%)
TYL-CHO-OVA	16	89	78	0	0	0
TYL-MA-OVA	0	0	0	0	0	0

^a^ Titer means the dilution of antiserum with an absorbance at 450 nm of about 1.0–1.5. ^b^ Inhibition = [(B − B_0_)/B] × 100%, under 1 μg/mL of competitor.

**Table 2:** Cross-reactivity (CR) of macrolides antibiotics or related compounds in the icELISA.

Compound	Structure	icELISA	
		IC_50_ (ng/mL)	CR * (%)
Tylosin	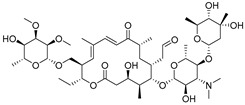	4.7	100
Tilmicosin	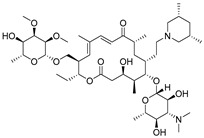	9.6	49.0
Erythromycin	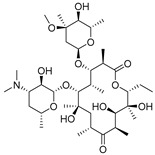	>2000	<0.1
Roxithromycin	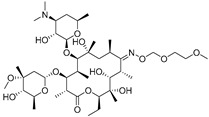	>2000	<0.1
Spiramycin	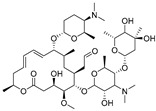	>2000	<0.1
Acetylspiramycin	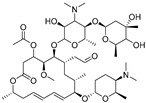	>2000	<0.1
Abamectin	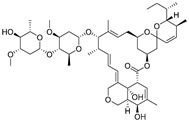	>2000	<0.1
Azithromycin	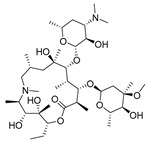	>2000	<0.1
Enrofloxacin	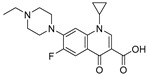	>2000	<0.1
Pefloxacin	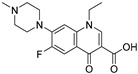	>2000	<0.1

* The values of CR were calculated by using the following equation: CR (%) = [IC_50_ (melatonin, mg/mL or ng/mL)/IC_50_ (cross-reactant, mg/mL or ng/mL)] × 100%.

**Table 3:** Characterization of mAbs against tylosin and cross-reaction with tilmicosin.

mAbs	Titer (10^3^)	IC_50_ (ng/mL, Tylosin)	IC_50_ (ng/mL, Tilmicosin)	Cross-React with Tilmicosin (%)
L01	1.6	6.2	16.1	38.5
L02	1.6	5.0	8.5	58.8
L03	1.6	5.2	9.2	56.4
L04	1.6	5.9	21.5	26.9

The mAbs were diluted 1:4000 in PBST (0.01 mol/L, pH 7.4) with 0.11 μg/mL of coating antigen.

**Table 4:** Recoveries of tylosin-spiked and tilmicosin-spiked testing by icELISA and HPLC-MS/MS (*n* = 3) ^a^.

			icELISA			HPLC-MS/MS		
Samples	Spiked	Spiked level (ng/mL)	Measured (ng/mL) (mean ± SD ^b^)	Recoveries (%)	CV (%)	Measured (ng/mL) (mean ± SD)	Recoveries (%)	CV (%)
Milk	Tylosin	25	21.6 ± 1.6	86.4	7.4	20.16 ± 0.41	80.6	2.0
		50	41.8 ± 6.0	83.5	14.4	34.15 ± 2.67	68.3	7.8
		100	88.4 ± 5.4	88.4	6.1	78.61 ± 0.71	78.6	0.9
	Tilmicosin	25	24.7 ± 2.2	98.7	8.9	28.67 ± 0.17	114.7	0.6
		50	54.8 ± 2.0	109.5	3.6	55.05 ± 0.71	110.1	1.3
		100	83.0 ± 3.5	83.0	4.2	114.35 ± 2.66	114.4	2.3
Drinking water	Tylosin	5	4.6 ± 0.2	92.2	4.3	3.61 ± 0.41	72.1	11.3
		10	7.6 ± 0.2	76.4	2.6	6.78 ± 0.91	67.8	13.4
		20	19.8 ± 0.6	99.2	3.0	14.52 ± 1.18	72.6	8.2
	Tilmicosin	5	4.4 ± 0.6	87.0	13.6	3.40 ± 0.27	67.9	7.9
		10	9.1 ± 0.1	91.1	1.1	6.65 ± 0.03	66.5	0.5
		20	17.2 ± 1.4	85.9	8.1	14.11 ± 0.81	70.6	5.7
Tap water	Tylosin	5	4.1 ± 0.1	81.9	2.4	5.44 ± 0.01	108.7	0.1
		10	8.3 ± 0.3	82.8	3.6	11.04 ± 0.72	110.4	6.6
		20	17.7 ± 0.7	88.2	4.0	22.85 ± 0.18	114.3	0.8
	Tilmicosin	5	4.2 ± 0.3	84.5	7.1	3.21 ± 0.14	64.3	4.3
		10	8.0 ± 1.1	80.0	13.8	6.65 ± 0.21	66.5	3.2
		20	18.8 ± 2.1	94.0	11.2	14.66 ± 0.45	73.3	3.1
Environmental water	Tylosin	5	3.9 ± 0.1	77.2	2.6	4.29 ± 0.13	85.9	3.0
		10	8.8 ± 0.3	87.9	3.4	9.57 ± 0.68	95.7	7.1
		20	17.0 ± 0.2	85.1	1.2	18.37 ± 1.36	91.8	7.4
	Tilmicosin	5	5.1 ± 0.1	101.7	2.0	3.24 ± 0.13	64.9	4.1
		10	7.9 ± 0.1	79.2	1.3	8.10 ± 0.30	81.0	3.7
		20	18.1 ± 1.2	90.3	6.6	15.20 ± 1.08	76.0	7.1

^a^ The spiked positive samples were tested per concentration by icELISA and HPLC-MS/MS. ^b^ Standard deviation.

**Table 5:** The detection amount of real positive samples (*n* = 3) ^a^.

Sample	Number	icELISA (ng/mL) (mean ± SD ^b^)	HPLC-MS/MS (ng/mL) (mean ± SD)	
		TYL/TMC	TYL	TMC
Milk	1	17.9 ± 0.9	18.86 ± 0.86	ND ^c^
	2	17.7 ± 0.4	20.58 ± 1.06	ND ^c^
	3	9.4 ± 0.9	9.21 ± 0.07	ND ^c^
Environmental water	4	0.5 ± 0.1	ND ^c^	1.35 ± 0.15
	5	0.7 ± 0.1	ND ^c^	1.84 ± 0.16
	6	0.9 ± 0.1	ND ^c^	1.83 ± 0.05
	7	1.2 ± 0.1	0.11 ± 0.01	3.18 ± 0.09
	8	8.3 ± 1.1	10.04 ± 0.23	ND ^c^
	9	6.2 ± 0.5	7.24 ± 0.42	ND ^c^
	10	4.6 ± 0.1	4.93 ± 0.13	2.27 ± 0.07
	11	18.1 ± 0.6	20.38 ± 0.61	ND ^c^

^a^ The positive samples were tested by icELISA and HPLC-MS/MS. ^b^ Standard deviation. ^c^ ND: Not detected.

## Supplementary Materials

The following are available online at https://www.mdpi.com/2218-273X/9/12/770/s1, Figure S1: The synthesis route of haptens, Figure S2: The full scan mass spectra of haptens, Figure S3: The UV-vis spectral data of conjugates, hapten and proteins, Figure S4: Optimization of icELISA working condition, Figure S5: The isotype of mAb L02, Figure S6: Mass spectra of mixture standard of tylosin and tilmicosin, Figure S7: The linear regression analysis of between icELISA with HPLC-MS/MS, Table S1: The result of chessboard method for icELISA.
