# Supplementary material for: Design of Novel Haptens and Development of Monoclonal Antibody-Based Immunoassays for the Simultaneous Detection of Tylosin and Tilmicosin in Milk and Water Samples
Source: Biomolecules. 2019 Nov 23;9(12):770. doi: 10.3390/biom9120770 (PMC6995535; doi:10.3390/biom9120770)
Supplement: Supplementary file 1 [file biomolecules-09-00770-s001.pdf]

supplementary materials

Desgin of Novel haptens and Development of Monoclonal Antibody-based Immunoassays for the Simultaneous Detection of Tylosin and Tilmicosin in Milk and Water Samples

Jian-xin Huang [1], Chan-yuan Yao [1], Jin-yi Yang [1], Zhen-feng Li [3], Fan He [1], Yuan-xin Tian [2,*], Hong Wang[1], Zhen-lin Xu [1], Yu-dong Shen [1,*]

[1] College of Food Science, Guangdong Provincial Key Laboratory of Food Quality and Safety, South China Agricultural University, Guangzhou 510642, Guangdong, P. R. China; bi@stu.scau.edu.cn (J-x.H.); ciel32o@163.com (C-y.Y.); yjy361@163.com (J-y.Y.); snowywhite@163.com (F.H.); gzwhongd@163.com (H.W.); jallent@163.com. (Z-l.X.)

[2] Guangdong Provincial Key Laboratory of New Drug Screening, School of Pharmaceutical Sciences, Southern Medical University, Guangzhou, 510515, Guangdong, P. R. China; tyx523@163.com (Y-x.T.)

[3] Department of Entomology and Nematology and UCD Comprehensive Cancer Center, University of California Davis, Davis, CA 95616, USA; fzhli@ucdavis.edu (Z-f.L.)

* Corresponding: shenyudong@scau.edu.cn (Y-d.S.), Tel: +86-20-85283448 (Y-d.S.); tyx523@163.com (Y-x.T.), Tel: +86-20-62789416 (Y-x.T.)

This material includes: The synthesis route of haptens (Figure S1), the full scan mass spectra of haptens (Figure S2), UV absorption curve of conjugates (Figure S3), optimization of icELISA working condition (Table S1, Figure S4), the isotype of mAb L02 (Figure S5), HPLC-MS/MS calibration (Figure S6), the linear regression analysis of between icELISA with HPLC-MS/MS (Figure S7).

Antigen preparation. The synthesis route and full scan mass spectra of hapten TYL-CHO was showed in figure S1, figure S2. Then the hapten were coupled to BSA as the immunogen and to OVA as the coating antigen by active ester method. The successful conjugations were confirmed by UV-visible spectra (Figure S3).

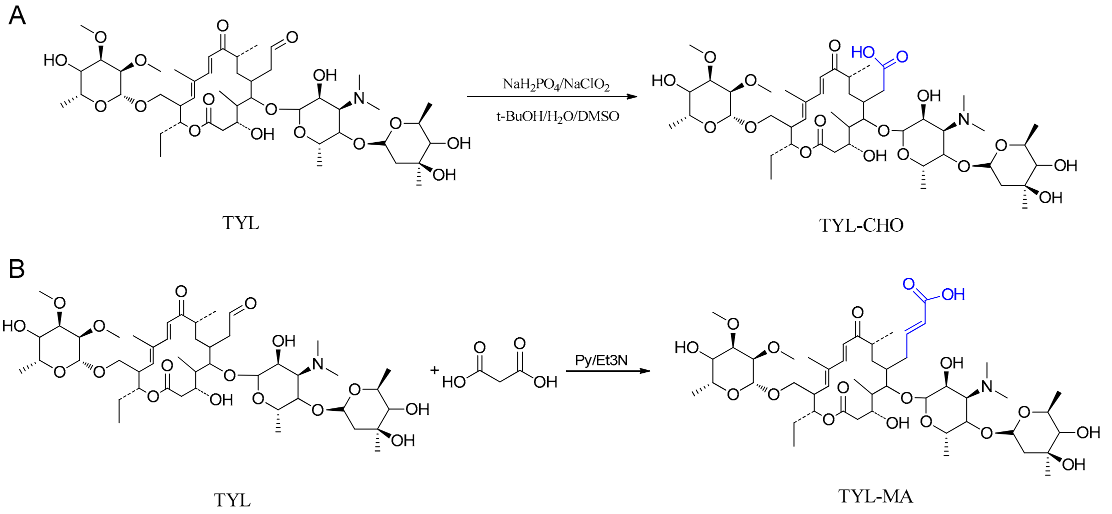

Figure S1. The synthesis route of haptens.

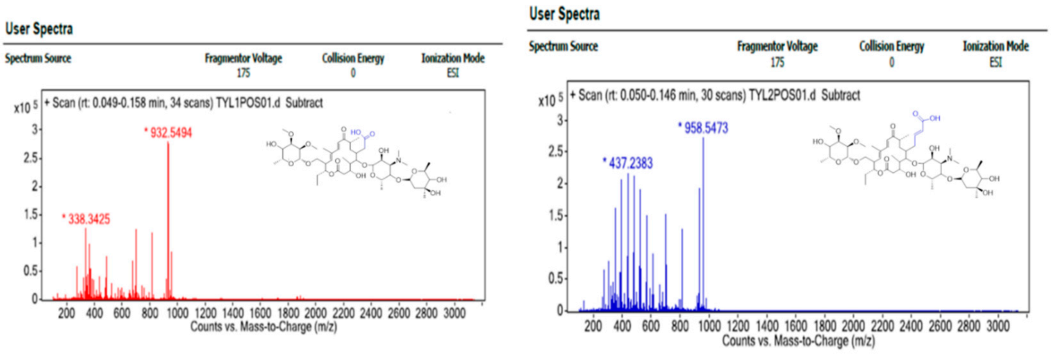

(A) (B)

Figure S2. Full scan mass spectra of four haptens. (A: TYL-CHO, [M+H]+ m/z 932.5494; B: TYL-MA, [M+H]+ m/z 957.53)

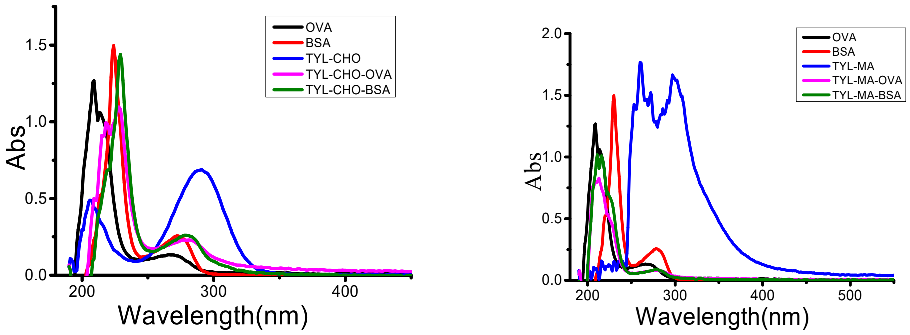

(A) (B)

Figure S3. The UV-vis spectral data of conjugates, hapten and proteins. (A: TYL-CHO; B: TYL-MA)

Optimization of icELISA working condition (Figure S4, Table S1). Several parameters in icELISA were optimized, including the concentration of antibody, coating antigens and HRP-IgG, working buffer (ionic strength, pH value and the concentration of tween-20) and reaction time. According to the chessboard titration and dose–response curves, the maximum absorbance value (Amax) and half maximal inhibitory concentration (IC50) were calculated. The optical conditions were confirmed by Amax, Amax/IC50 and IC50. A condition with higher value of Amax/IC50 and lower value of IC50 were selected to use in assay.

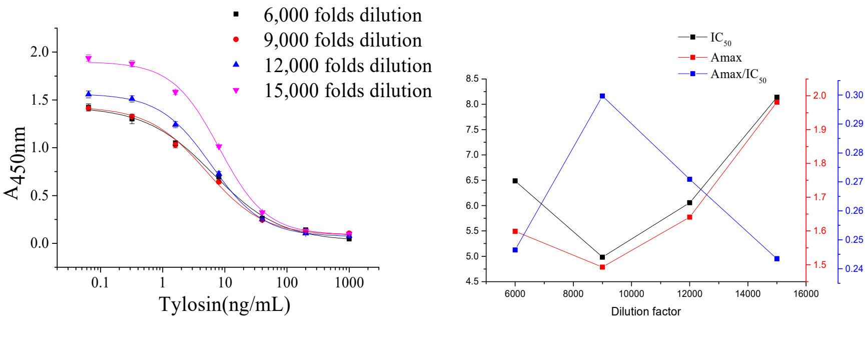

(A) (B)

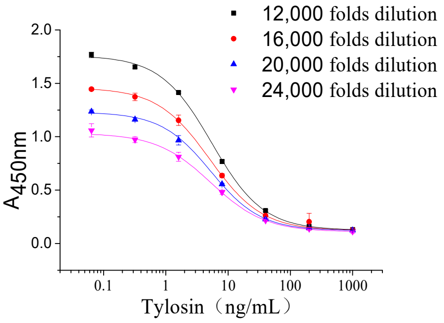
12,000 folds dilution
16,000 folds dilution
20,000 folds dilution
24,000 folds dilution
A450nm
Tylosin（ng/mL）

(C)

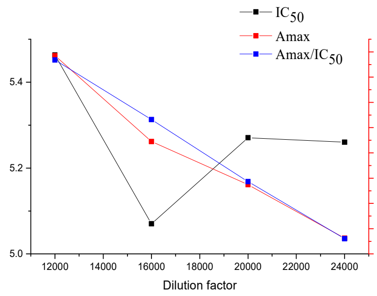
IC50
Amax
Amax/IC50
Dilution factor

(D)

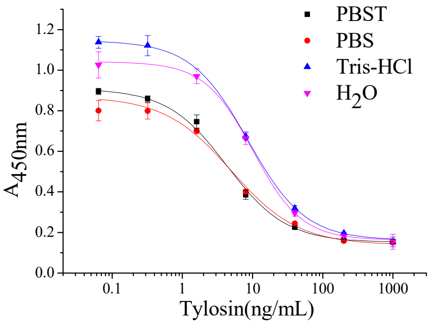
PBST
PBS
Tris-HCl
H2O
A450nm
Tylosin(ng/mL)

(E)

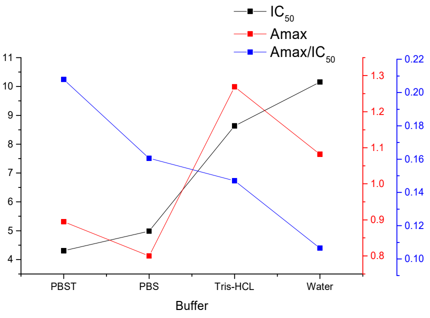
IC50
Amax
Amax/IC50
PBST
PBS
Tris-HCL
Water
Buffer

(F)

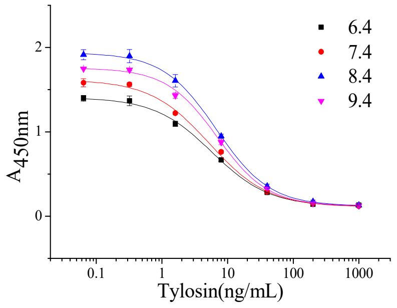
6.4
7.4
8.4
9.4
A450nm
Tylosin(ng/mL)

(G)

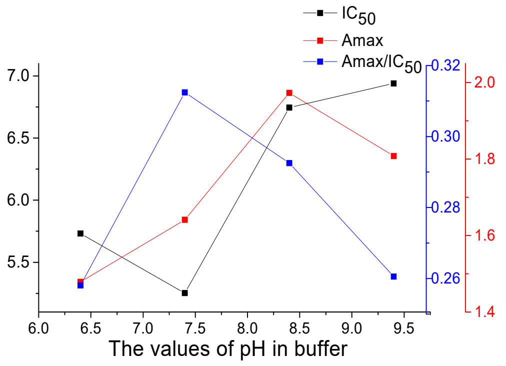
IC50
Amax
Amax/IC50
The values of pH in buffer

(H)

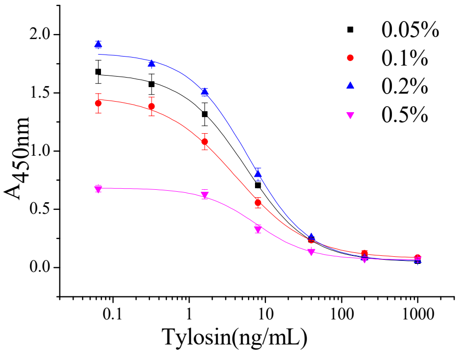
0.05%
0.1%
0.2%
0.5%
A450nm
Tylosin(ng/mL)

(I)

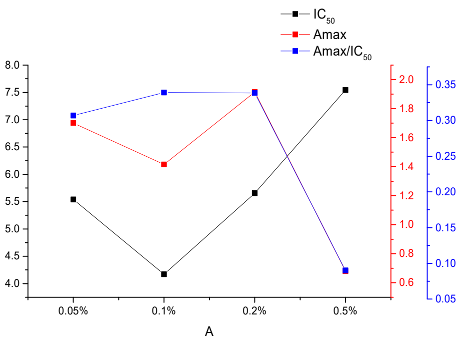
IC50
Amax
Amax/IC50
0.05%
0.1%
0.2%
0.5%
A

(J)

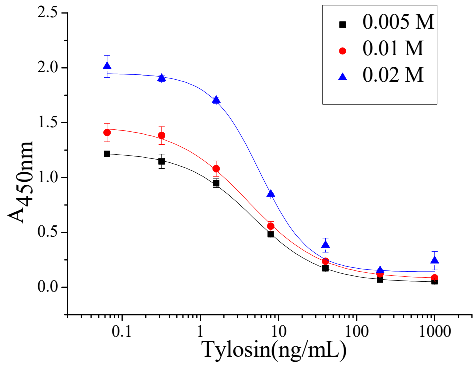

0.005 M
0.01 M
0.02 M
A450nm
Tylosin(ng/mL)

(K)

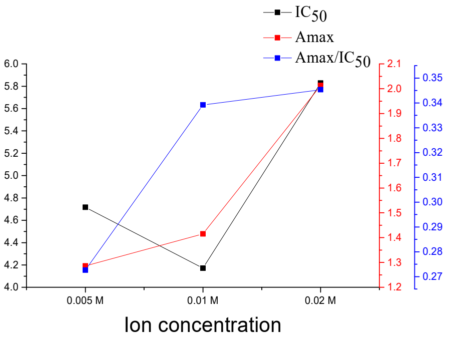

IC50
Amax
Amax/IC50
0.005 M
0.01 M
0.02 M
Ion concentration

(L)

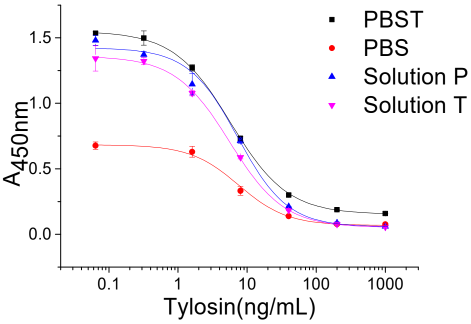

PBST
PBS
Solution P
Solution T
A450nm
Tylosin(ng/mL)

(M)

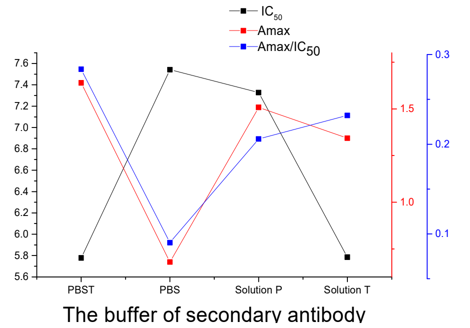

IC50
Amax
Amax/IC50
PBST
PBS
Solution P
Solution T
The buffer of secondary antibody

(N)

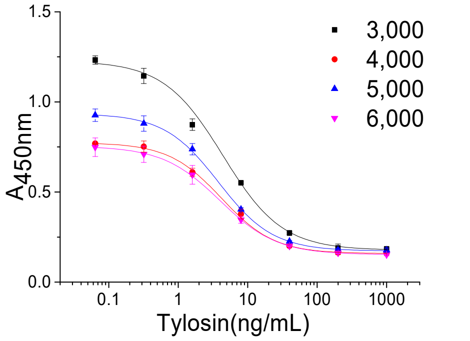

3,000
4,000
5,000
6,000
A450nm
Tylosin(ng/mL)

(O)

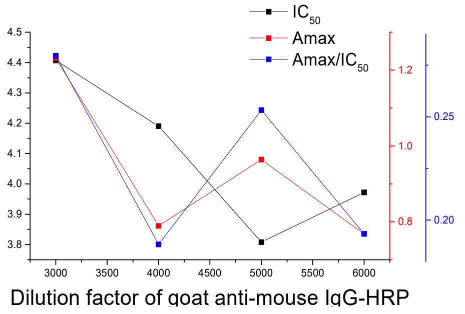

IC50
Amax
Amax/IC50
Dilution factor of goat anti-mouse IgG-HRP

(P)

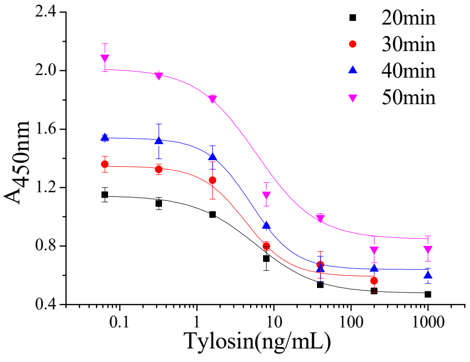

20min
30min
40min
50min
A450nm
Tylosin(ng/mL)

(Q)

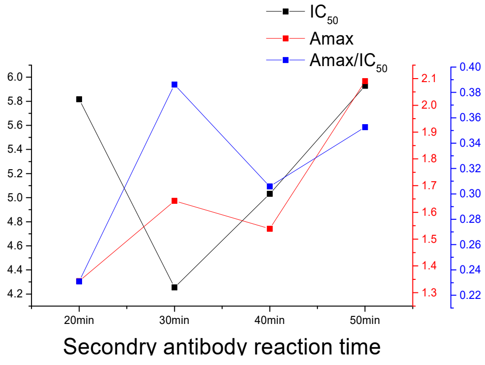

IC50
Amax
Amax/IC50
20min
30min
40min
50min
Secondry antibody reaction time

(R)

Figure S4. Optimization of icELISA working condition. Concentration of coating antigen (A, B), concentration of antibody (C, D), working buffer (E, F), pH of working buffer (G, H), Tween-20 content of buffer (I, J), ion concentration of buffer (K, L), the buffer of secondary antibody (M, N), dilution factor of goat anti-mouse IgG-HRP (O, P), secondary antibody reaction time (Q, R). A, C, E, G, I, K, M, O, Q are calibration curves, and B, D, F, H, J, L, N, P, R are trend curves of A_{MAX}, IC_{50} and A_{MAX}/IC_{50}.

The isotype of mAb L02 (Figure S5). The mAb L02 was diluted as 100 ng mL^{-1} by sample diluent. The commercial strip showed that multiple bands detected but the darkest band was the isotype of the most abundant antibody in the sample.

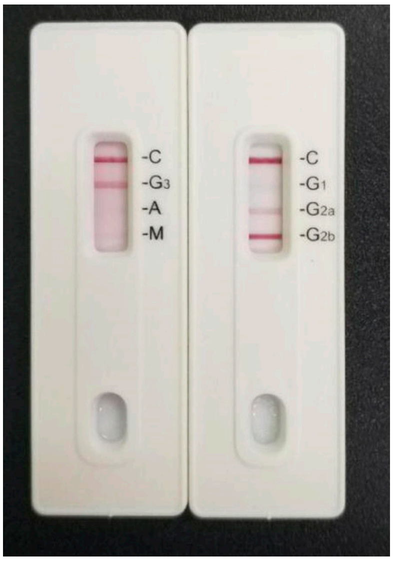

Figure S5. The isotype of mAb L02

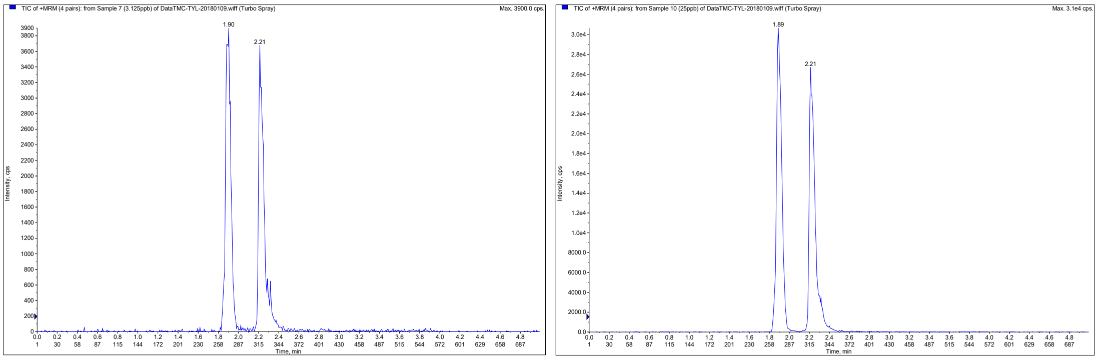

(A) (B)

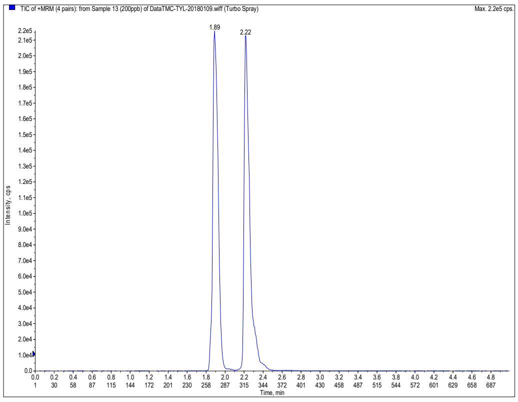

(C)

Figure S6. Mass spectra of mixture standard of tylosin and tilmicosin. (A, B, C: the concentration of mixture standard was 3.125 ng mL^{-1}, 25 ng mL^{-1}, 200 ng mL^{-1}.)

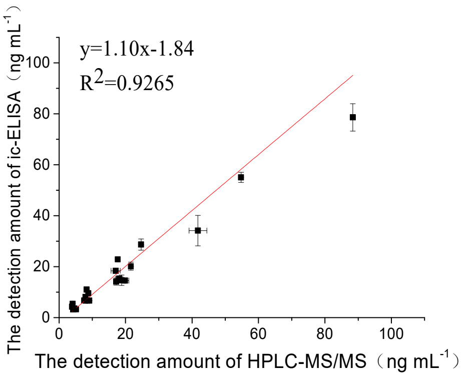

Figure S7. The linear regression analysis of between icELISA with HPLC-MS/MS

Table S1. The result of chessboard method for icELISA.

Dilution of antiserum	Dilution of coating antigen			
	6,000	9,000	12,000	18,000
1,000	3.42	3.42	3.08	2.10
2,000	3.15	2.94	2.37	1.53
4,000	3.01	2.45	1.68	**0.98**
8,000	2.58	1.62	**1.00**	0.56
16,000	1.69	**0.95**	0.56	0.33
32,000	**1.06**	0.53	0.33	0.20
64,000	0.61	0.33	0.20	0.13
0	0.08	0.06	0.06	0.07
